# Prolactin inhibits the progression of intervertebral disc degeneration through inactivation of the NF-κB pathway in rats

**DOI:** 10.1038/s41419-017-0151-z

**Published:** 2018-01-24

**Authors:** Xiexing Wu, Yu Liu, Xiaobin Guo, Wei Zhou, Liangliang Wang, Jiawei Shi, Yunxia Tao, Mo Zhu, Dechun Geng, Huilin Yang, Haiqing Mao

**Affiliations:** 1 Department of Orthopedics, The First Affiliated Hospital of Soochow University, Suzhou, Jiangsu China; 2 Department of Radiology, The First Affiliated Hospital of Soochow University, Suzhou, Jiangsu China

## Abstract

Intervertebral disc degeneration (IVDD) is one of the key predisposing factors for low back pain. Although the exact mechanism remains unclear, inflammatory response and nucleus pulposus (NP) apoptosis are known to play important roles in this process. Prolactin protects against inflammation-associated chondrocyte apoptosis in arthritis. Based on prior studies, we hypothesized that prolactin might have therapeutic effects on IVDD by inhibiting the apoptosis of degenerative human disc NP cells. An experimental model of IVDD was established in 3-month-old Sprague-Dawley rats by submitting them to percutaneous disc puncture with a 20-gauge needle on levels 7–8 and 8–9 of the coccygeal vertebrae. Then the rats were injected with 20 or 200 ng prolactin on a weekly basis. Radiologic and histologic analyses were performed on days 4, 7, 14, and 28. The expression of prolactin and its receptor was analyzed in human tissue obtained from symptomatic patients undergoing microencoscopy discectomy, or from scoliosis patients undergoing deformity correction surgery. The results showed that intradiscal injection of prolactin maintained disc height and the mean signal intensity of the punctured disc. Histological analysis indicated that prolactin treatment significantly retained the complete structure of the NP and annulus fibrosus compared with the vehicle group. In addition, more collagen II, but fewer collagen I-containing tissues were detected in the prolactin treatment groups compared to the vehicle group. Moreover, low levels of tumor necrosis factor-α, interleukin-1β, cleaved-caspase 3, and TUNEL staining were observed in the prolactin treatment groups. We also demonstrated that prolactin impaired puncture-induced inflammation and cell apoptosis by downregulating activation of the NF-κB pathway. The degenerated NP tissues from patients had decreased expression of prolactin and its receptor, whereas expression was increased in the NP tissues removed from scoliosis patients. These results suggest that prolactin may be a novel therapeutic target for the treatment of IVDD.

Low back pain has become a serious public health problem in modern society^1^, and has imposed huge psychological, physiological, and economic burdens^2^. Among all of the predisposing factors, intervertebral disc degeneration (IVDD) is one of the most important and prevalent^3^. The intervertebral disc (IVD) consists of three components: a central nucleus pulposus (NP), a peripheral annulus fibrosus (AF), and two vertebral endplates^4^. In general, the gelatinous NP is the main functional composition of IVD, and degeneration of the NP is regarded as a crucial part of IVDD^5^.

Although the pathogenesis of IVDD is complicated and not well defined, it is thought that inflammation and NP cell (NPC) apoptosis might play critical roles in this condition^3^. NPCs are the main type of cells resident in NP, which produce extracellular matrix (ECM) including collagen II and proteoglycans, which are the main components of the gelatinous tissues of NP. Studies have shown that the upregulation of inflammatory cytokines, especially tumor necrosis factor-α (TNF-α) and interleukin-1β (IL-1β), contribute to the acceleration of IVDD by inducing aggrecan degradation^5^. In addition, the apoptosis of NPCs may lead to the remodeling of different cell types and a reduction in synthesis of the ECM, thereby maintaining the stability of the IVD^6^. Zhang et al.^7^ demonstrated that inhibiting the apoptosis of cells in the IVD attenuated its degenerative progression in a rat model. Thus, reducing the inflammatory response and decreasing the apoptosis of NPCs are considered to be suitable ways to treat IVDD.

Prolactin (PRL), a circulating protein hormone secreted by acidophilic cells in the anterior pituitary^8^, regulates differentiation and lactation in the mammary epithelium^9^, and also plays an important role in regulating numerous functions such as inflammation, cell survival, and proliferation^10,11^ by binding to the PRL receptor^12^. Hartwell et al.^13^ reported that PRL inhibited TNF-α and IL-1β expression in mice. In addition, several authors have demonstrated the protective effects of PRL against chondrocyte apoptosis^14,15^. Although it is known that NPCs consist of notochord cells and chondrocyte-like cells^16^, little is known about how PRL is regulated in NPCs. Considering the important role of the inflammatory response and NPC apoptosis in IVDD, we hypothesized that PRL might also play a role in this condition.

In this study, we determined if PRL and its receptor are expressed in NP tissues. In addition, we used a 33-gauge needle to perform intradiscal injection of different concentrations of exogenous PRL into the NP of rats with IVD injury, to evaluate the effects on IVDD. Finally, we explored the possible mechanisms underlying the effects of PRL on IVDD.

## Results

### Low expression of PRL and its receptor in degenerated NP tissues

**Fig. 1:**
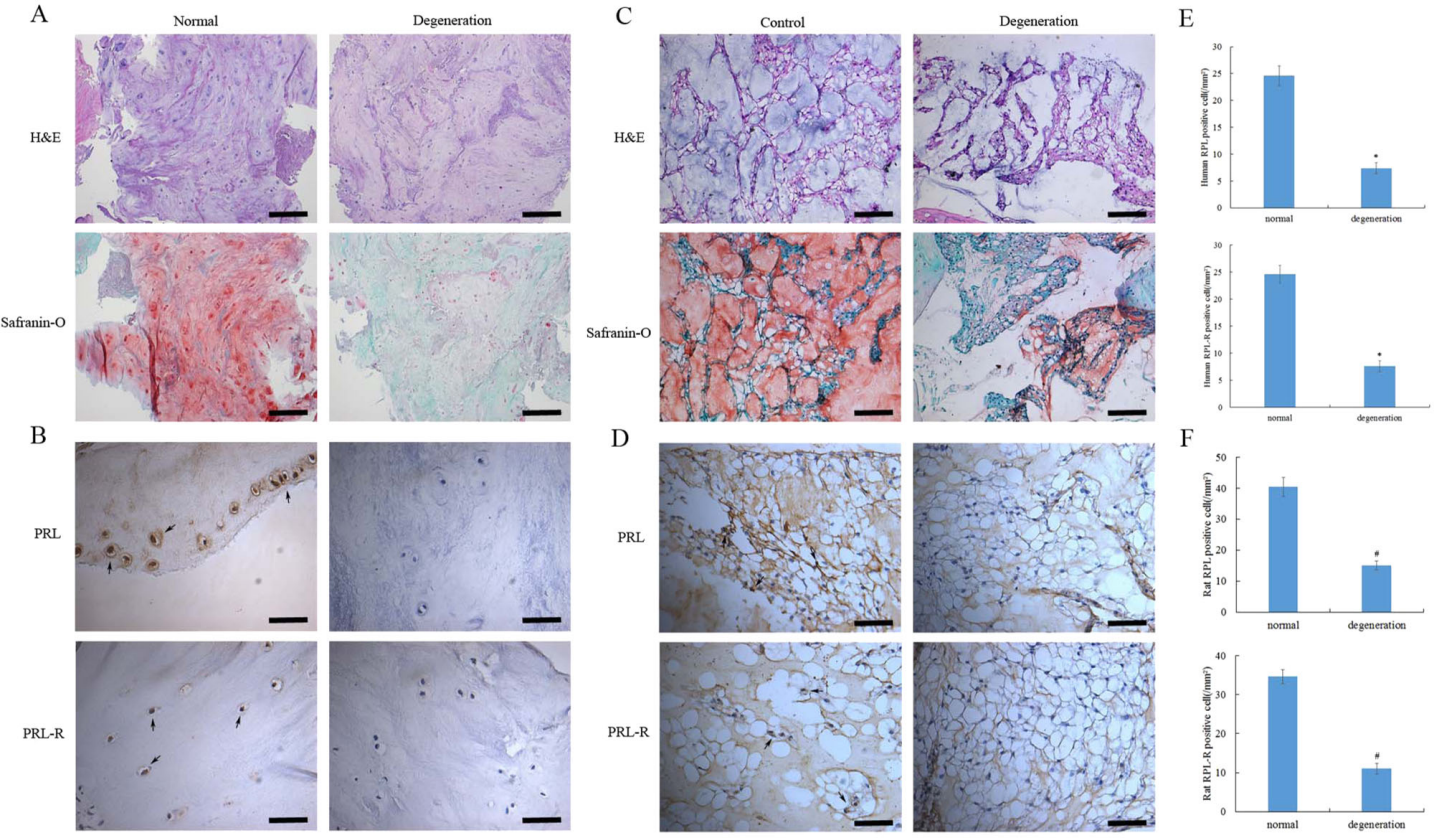
Degenerated NP tissues had low expression of PRL and its receptor **a** H&E and Safranin-O staining of human NP tissues (Scar bar = 500 μm). **b** Immunohistochemical staining of PRL and its receptor in human NP tissues (Scar bar = 50 μm). **c** H&E and Safranin-O staining of rat NP tissues (Scar bar = 100 μm). **d** Immunohistochemical staining of PRL and its receptor in rat NP tissues (Scar bar = 50 μm). **e**, **f** Quantification of PRL and its receptor in the NP tissues of humans and rats. ^*^P < 0.05 vs. the normal group, ^#^*P* < 0.05 vs. the control group

To investigate whether PRL may play a role in IVDD, we first evaluated the expression of PRL and its receptor in NP tissues. Immunohistochemical staining showed less expression of PRL and its receptor in human degenerated NP tissues compared to non-degenerated tissues (Fig. 1a, b). We established a rat IVDD model by needle puncture of the IVD. Hematoxylin and eosin (H&E) staining showed a decrease in the NPCs and ECM, meanwhile less staining Safranin-O indicated the absence of collagens and aggrecans in the degeneration group (Fig. 1c). Immunohistochemical staining showed significantly lower levels of PRL and its receptor in rat degenerated NP tissues compared to the control group, which was consistent with the results obtained from human tissues (Fig. 1d). Quantification of the expression of PRL and its receptor in NP tissues from both humans and rats exhibited a decrease in PRL-positive cells in the degeneration group (Fig. 1e, f). These results revealed the presence of PRL and its receptor in NP tissues.

### PRL retards IVDD in a rat model

**Fig. 2:**
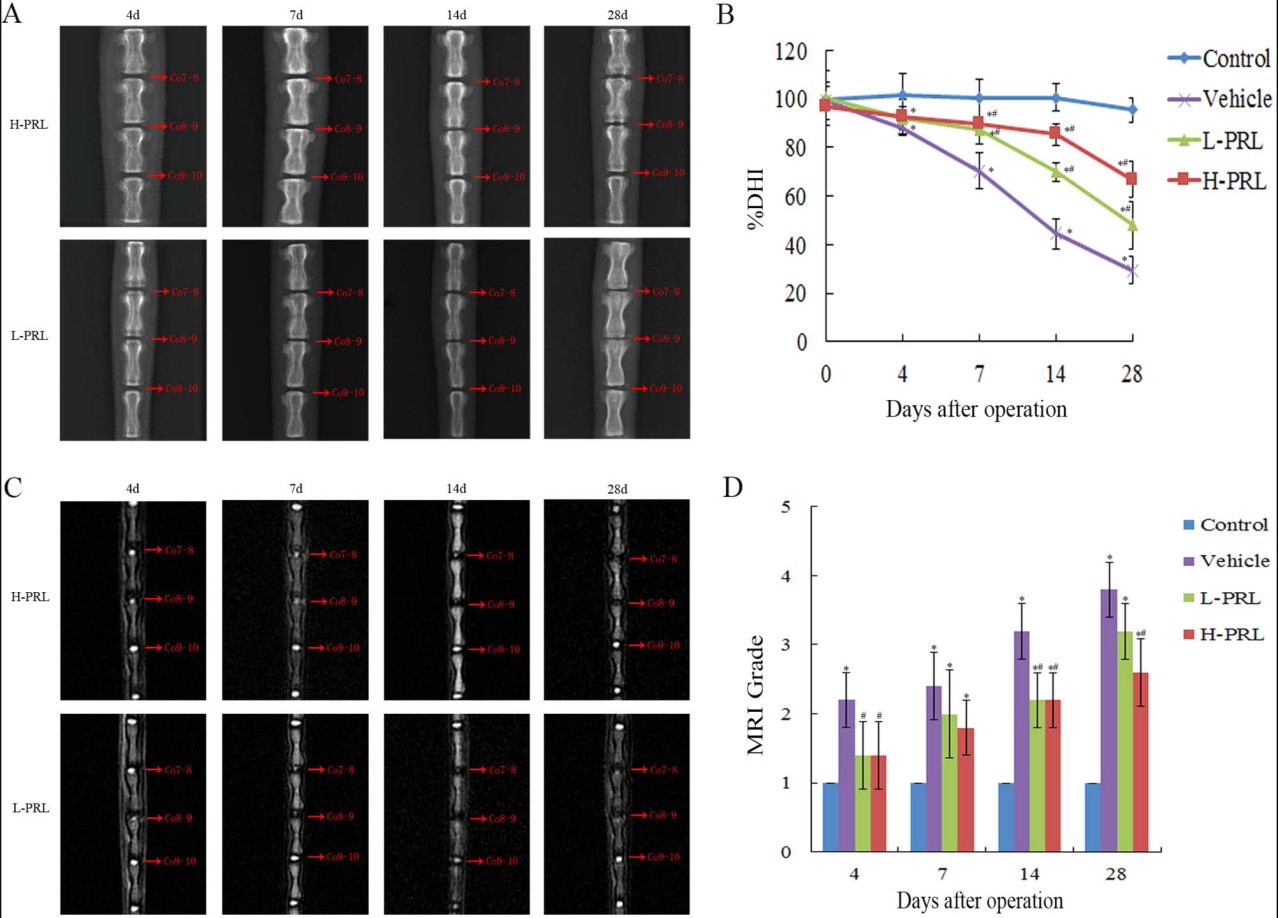
Imaging and analysis on days 4, 7, 14, and 28 after intradiscal injection of exogenous PRL in a rat IVDD model induced by needle puncture **a** Typical radiographs of coccygeal vertebrae on days 4, 7, 14, and 28 after initial puncture. **b** Sequential changes in DHI with time in all of the groups. **c** Representative MRI scans of coccygeal vertebrae on days 4, 7, 14, and 28 after initial puncture. **d** Changes in MRI grade with time in all of the groups. ^*^P < 0.05 vs. the control group, ^#^*P* < 0.05 vs. the vehicle group

**Fig. 3:**
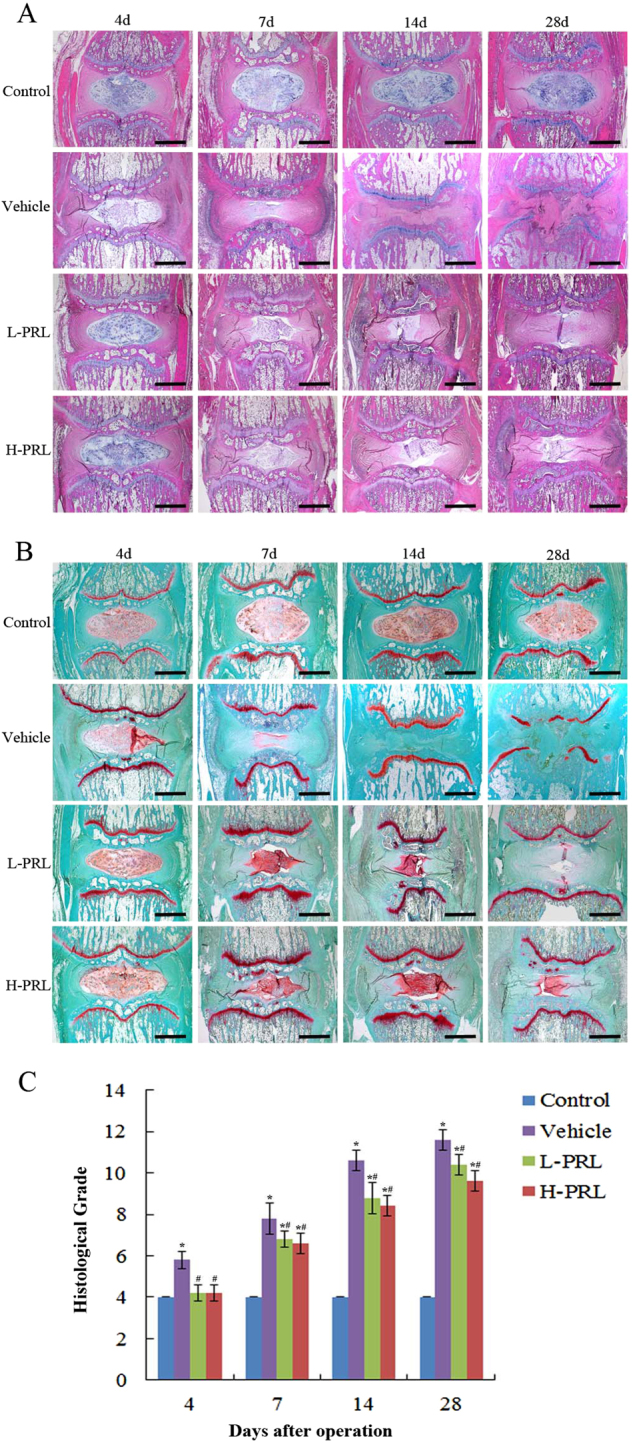
Histological staining and histomorphometric analysis of the rat coccygeal IVD **a** H&E staining. **b** Safranin-O staining. **c** Histological scores on days 4, 7, 14, and 28 after initial puncture. Scar bar = 1000 μm. ^*^P < 0.05 vs. the control group, ^#^*P* < 0.05 vs. the vehicle group

**Fig. 4:**
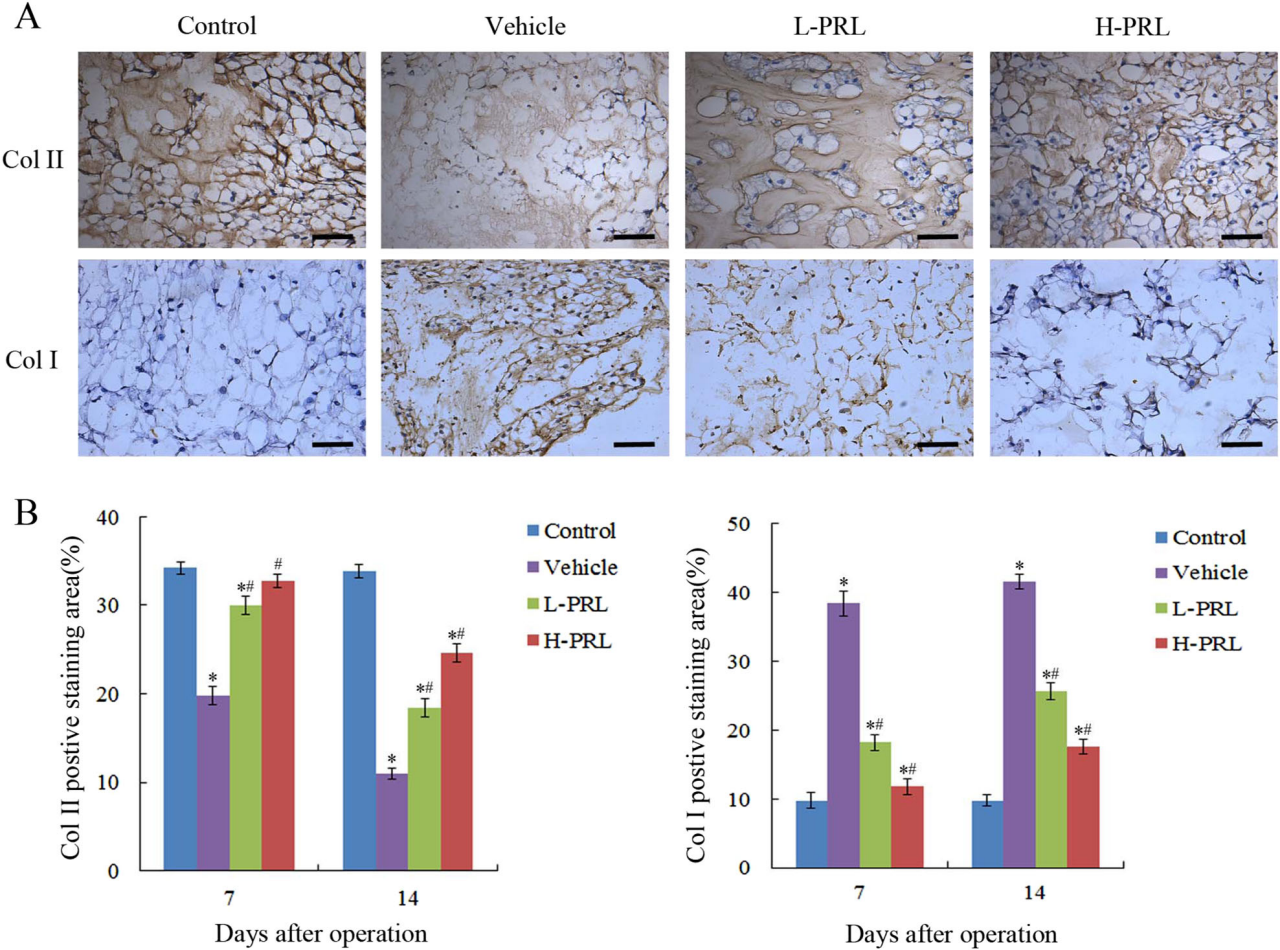
PRL treatment reduced remodeling of the ECM **a** Immunohistochemical staining of collagen II and collagen I in intervertebral NP tissues of the rat 7 d after initial puncture. **b** Semi-quantification of collagen II and collagen I staining on days 7 and 14 after initial puncture. Scar bar = 50 μm. ^*^P < 0.05 vs. the control group, ^#^*P* *<* 0.05 vs. the vehicle group

To assess the therapeutic effects of PRL in vivo, intradiscal injection of exogenous PRL was performed weekly in a rat IVDD model induced by needle puncture. The X-ray results at 4 d showed no significant distinction in disc height between rats with punctured and non-punctured discs (*P* > 0.05). At 7 d after initial puncture, the phosphate-buffered saline (PBS) injection revealed more significant narrowing of disc height compared with non-surgical levels. Interestingly, the decline in disc height began to slow down after PRL treatment (Fig. 2a), and the percent disc height index (%DHI) in the low- and high-concentration PRL treatment groups was 1.24- and 1.28-fold higher than that in the PBS injection group (Fig. 2b). According to the magnetic resonance imaging (MRI) results, 4 d after initial annulus puncture, the discs injected with PBS had a weaker MRI signal than the non-punctured discs, and the differences became more apparent over time. PRL treatment efficiently alleviated the weak MRI signal intensity in the punctured disc, whereas the MRI signal significantly declined compared with PBS injection disc levels (Fig. 2c, d). The histological structure of the IVD can was clearly observed by histological staining. Compared with the control group, the vehicle group exhibited a significant decrease of NPCs and destruction of AF lamella, which was particularly evident on day 28 (Fig. 3a, b). PRL treatment significantly retained the complete structure of the NP and AF compared with the vehicle group, and the histological grade of the low- and high-PRL groups decreased by 12.8% (*P* < 0.05) and 15.4% (*P* < 0.05), respectively, compared with the vehicle group (Fig. 3c). Furthermore, immunohistochemical staining showed intense collagen II expression in the control group but little expression in the vehicle group, indicating more ECM loss in the vehicle group. In contrast, the vehicle group had greater collagen I expression than the control group, indicating an increase in fibrosis and ECM proteins, which contribute to IVDD^17^. However, PRL treatment significantly increased and decreased the expression of collagen II and collagen I, respectively, compared with the vehicle group (Fig. 4). These results suggest that exogenous PRL treatment can significantly alleviate the progression of IVDD.

### PRL downregulates TNF-α and IL-1β expression

**Fig. 5:**
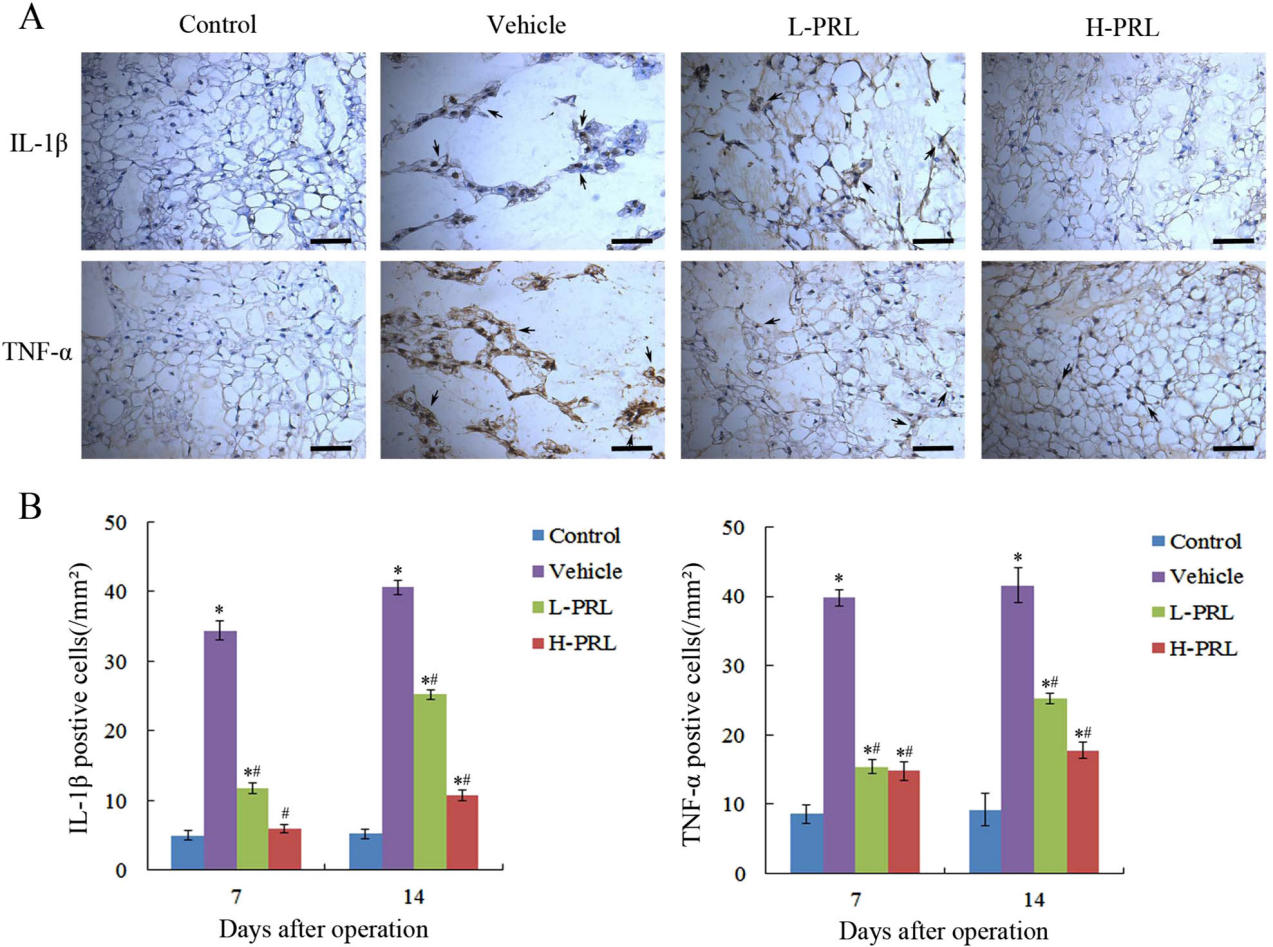
PRL inhibited the expression of inflammatory cytokines **a** Immunohistochemical staining of TNF-α and IL-1β in the NP in vivo. **b** Quantification of TNF-α and IL-1β staining on days 7 and 14 after initial puncture. Scar bar = 50 μm. ^*^P < 0.05 vs. the control group, ^#^*P* < 0.05 vs. the vehicle group

TNF-α and IL-1β play important roles in IVDD, thus we validated the inhibitory effects of PRL on inflammation in a rat IVDD model. Immunohistochemical staining showed high TNF-α and IL-1β expression in the PBS injection group, and statistical analysis showed that needle puncture induced degeneration of NP, which led to 4.6-fold and 6.8-fold higher TNF-α and IL-1β expression, respectively, compared to the control group, whereas PRL treatment significantly downregulated their expression. Specifically, in the low- and high-PRL treatment groups, the number of TNF-α-positive cells decreased by 61.3% (*P* < 0.01) and 62.8% (*P* < 0.01), respectively, and the number of IL-1β-positive cells decreased by 65.7% (*P* < 0.01) and 82.6% (*P* < 0.01), respectively, compared with the PBS injection group. These results showed that exogenous PRL treatment inhibited the inflammatory response in vivo (Fig. 5).

### PRL prevents the apoptosis of NPCs

**Fig. 6:**
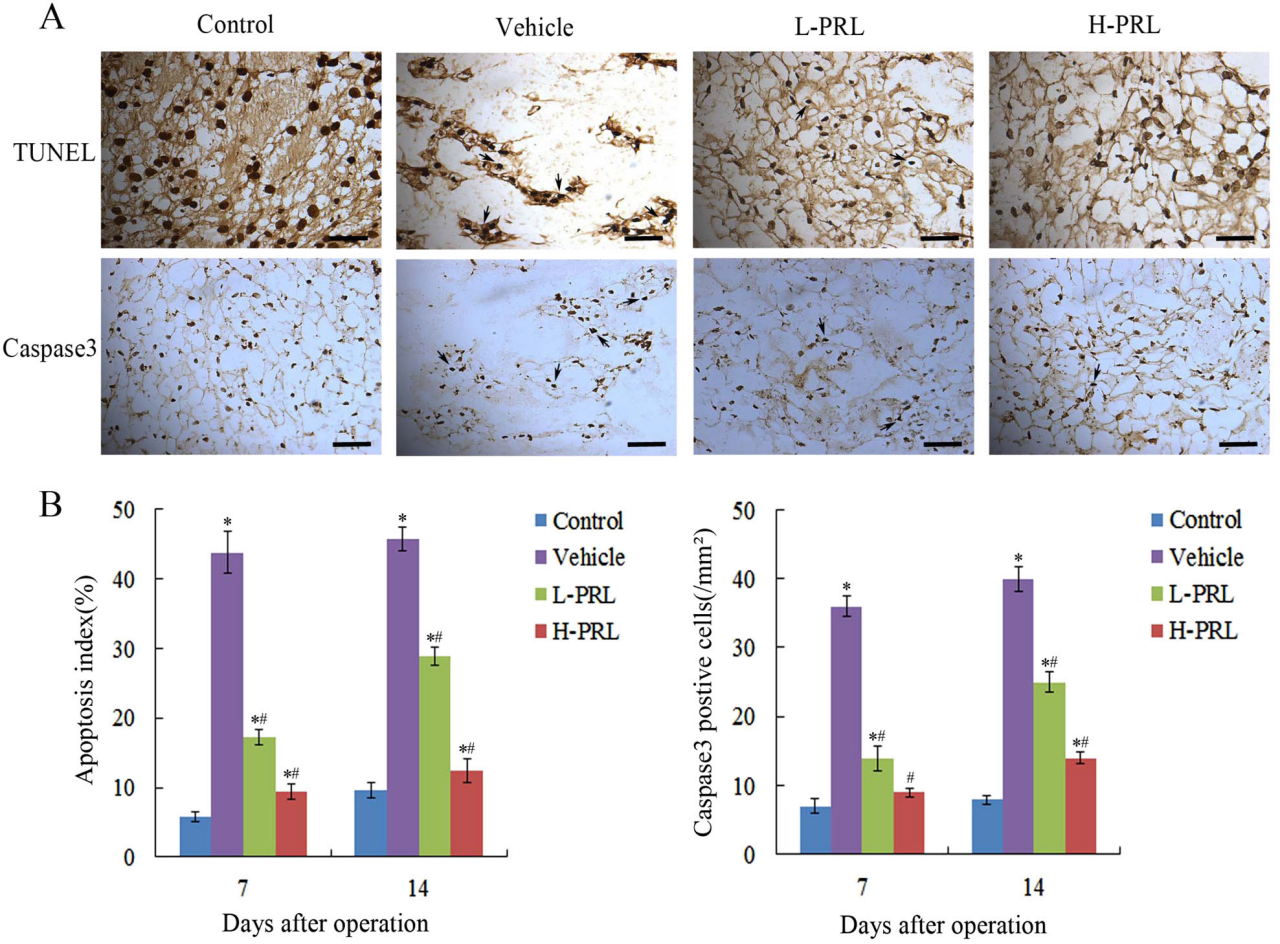
PRL and apoptosis of NPCs in vivo **a** TUNEL staining and staining of cleaved caspase 3 in the NP in vivo. **b** Semi-quantification of apoptotic index and quantification of cleaved caspase 3 staining on days 7 and 14 after initial puncture. Scar bar = 50 μm. ^*^P < 0.05 vs. the control group, ^#^*P* < 0.05 vs. the vehicle group

We used terminal deoxynucleotidyl transferase (TdT) dUTP nick-end labeling (TUNEL) staining to evaluate the apoptosis of NPCs. Percutaneous disc puncture with a 20-gauge needle significantly increased apoptosis in the disc compared with the control group. By contrast, fewer TUNEL-positive cells were detected in the PRL treated groups. The apoptotic index showed a significant decrease by 60.7% (*P* < 0.01) in the low-PRL treatment group and by 78.5% (*P* < 0.01) in the high-PRL treatment group, compared with the vehicle group. Additionally, immunohistochemical staining showed more cleaved caspase 3-positive cells in the degeneration group than in the control group. PRL injection significantly decreased cleaved caspase 3 expression in the low- and high-PRL treatment groups by 61.1 and 75% (*P* < 0.01), respectively. These data were in accordance with the results of the TUNEL assay, which verified the antagonistic effects of PRL on apoptosis (Fig. 6).

### PRL inhibits activation of the NF-κB pathway

**Fig. 7:**
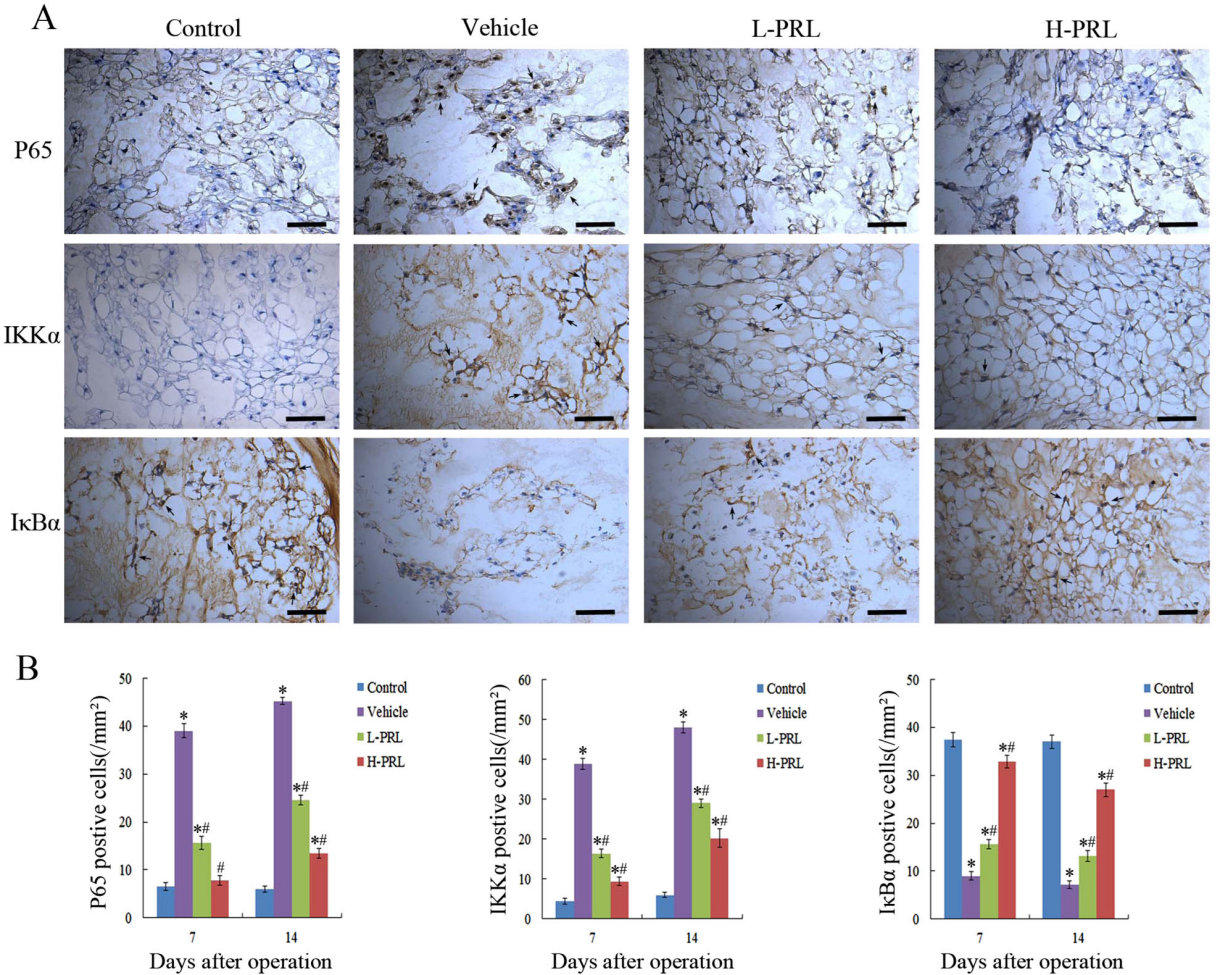
PRL treatment decreased activation of the NF-κB pathway **a** Immunohistochemical staining of p65, IKKα, and IκBα in intervertebral NP tissues of rats 7 d after initial puncture. **b** Quantification of p65, IKKα, and IκBα on days 7 and 14 after initial puncture. Scar bar = 50 μm. ^*^P < 0.05 vs. the control group, ^#^*P* < 0.05 vs. the vehicle group

To understand the mechanisms underlying prevention of IVDD by PRL, we evaluated the activity of the NF-κB signaling pathway, which plays a major role in the pathogenesis of IVDD. The expression of p65 and IκB kinase α (IKKα) significantly increased, whereas inhibitor of NF-κBα (IκBα) expression decreased in the degeneration group, which indicated activation of the NF-κB pathway during IVDD. PRL treatment effectively inhibited activation of NF-κB, the number of p65-positive cells decreased by 60 and 80%, and IKKα decreased by 57.7 and 75.8% (*P* < 0.01), in contrast to IκBα, which increased by 1.7-fold and 3.6-fold (*P* < 0.01) in the low- and high-PRL treatment groups, respectively, compared with the degeneration group. These results suggest that PRL may retard the progression of IVDD by downregulating NF-κB activity (Fig. 7).

## Discussion

Due to the high morbidity associated with IVDD and the limitation of current treatment modalities^18^, novel approaches with the potential to retard or even reverse disc degeneration and restore physiologic disc function are urgently needed. A large number of studies have reported the therapeutic effects of some biological agents in this condition^19–21^, but few of these drugs are currently applied in clinical practice. Our study demonstrated for the first time that the hormone secreted by human body, PRL, might play a role in IVDD. Furthermore, our results showed that IVDD could be retarded by intradiscal injection of exogenous PRL by needle puncture of the IVD. Radiographic examination showed that PRL treatment resulted in the maintenance of intervertebral disc height and T2-weighted signal intensity. These results confirmed the therapeutic effects of PRL in IVDD.

PRL, a pituitary peptide hormone, was first discovered for its ability to stimulate the proliferation and differentiation of the mammary cells required for lactation. In the past decade, many new insights into the functions of PRL and its receptor have been revealed^22^. According to recent studies, PRL has more than 300 functions, which are not merely physiological effects like reproduction, lactation, growth, and metabolism, but although functions of promoting proliferation, protecting against apoptosis, and enhancing cell survival. All of these actions are mediated by its transmembrane receptor, the PRL receptor, which is ubiquitously expressed^23^. In this study, we found that PRL and its receptor had less expression in degenerated NP tissues than in normal ones, and the degree of degeneration could be alleviated by intradiscal injection of exogenous PRL. Thus, it appears that PRL and its receptor are expressed in the NP, and might play a role in slowing down IVDD. Nonetheless, the mechanism underlying the role that PRL plays in retarding IVDD is not clearly understood.

IVDD is a multifactorial disease, involving the production of pro-inflammatory cytokines, apoptosis of NPCs, degradation of the ECM, and nutrient deprivation^24^. Pro-inflammatory cytokines, especially TNF-α and IL-1β, play important roles in IVDD^25^. There is accumulating evidence that IL-1β is capable of upregulating zinc-based matrix degrading enzymes, in particular matrix metalloproteinases, disintegrins, and metalloproteinases with thrombospondin motif, which may lead to degradation of collagen II and proteoglycan, as a result of losing ECM and severely reducing the ability of the NP to retain water^26^. TNF-α has been implicated in disc herniation and nerve root pain^27^. Teixeira et al. showed a new therapeutic method for IVD degeneration by diclofenac-nanoparticles intradiscal injection, based on the modulation of inflammation^28^. In this study, we demonstrated that compared with the PBS injection group, immunohistochemical staining showed a decrease of TNF-α and IL-1β expression with PRL injection, indicating that PRL could suppress the inflammatory response during IVDD.

The apoptosis of NPCs during IVDD may cause additional degeneration. Gruber and Hanley reported that apoptotic cells play key roles in the pathogenesis of IVDD^29^, namely transformation of the microenvironment, nutrient depletion, and NPC stress during IVD degeneration. Chen et al.^30^ showed that metformin ameliorated disc degeneration by protecting NPCs against apoptosis. In our study, TUNEL and immunohistochemical staining showed that there were less apoptotic cells and less cleaved caspase 3, which acts as an initiation factor for apoptosis, after PRL treatment compared with the degeneration group. These results demonstrate the protective effects of PRL on apoptosis of NPCs during IVDD.

NF-κB is a family of transcription factors that plays a central role in mediating the cellular response to damage, stress, and inflammation^31^. Its activation leads to the upregulation of pro-inflammatory cytokines and matrix-degrading enzymes in IVDD. Moreover, inhibition of the NF-κB pathway can inhibit IL-1β-induced upregulation of MMPs and ADAMTS as a consequence of the reduced degradation of collagen II and proteoglycan^32^. The NF-κB pathway is also considered to be relevant to apoptosis in NPCs. Kang et al.^33^ reported that microRNA-494 enhanced apoptosis of degenerative human NPCs regulated by NF-κB. According to those previous studies, we investigated whether the functions of PRL were associated with the NF-κB pathway. Our results showed that NF-κB was significantly activated in the degeneration group, which is in line with the results of previous studies. In addition, when PRL was injected, there was an obvious decrease in p65- and IKKα-positive cells, and an increase in IκBα-positive cells. These results indicate that PRL can inhibit activation of the NF-κB pathway in IVDD.

The most prevalent form of NF-kB is a heterodimer consisting of p50 and p65, which typically exists in the cytoplasm with no transcriptional activity or binding of IκB proteins. When IκB is phosphorylated by IKK, it dissociates from NF-κB, allowing its translocation to the nucleus and the initiation of transcription^34,35^. Thus, inhibition of IKK or stabilization of IκB may be the targets for blocking NF-κB activity. To further investigate the relationship between PRL and the NF-kB pathway, we performed immunohistochemical staining to detect changes in IκBα and IKKα expression. The activation of IKKα was suppressed by PRL in a dose-dependent manner as a result of decreased IκBα phosphorylation, indicating that PRL inhibited activation of the NF-κB pathway via suppressing the IKKα–IκBα interaction. However a previous study presented contrary results. Olavarría et al.^36^ showed that PRL induced the production of reactive oxygen species and the expression of TNF-α and IL-1β in bony fish via the NF-κB signaling pathway. Thus, there may be different effects in different species and tissues, which should be confirmed by additional studies.

In summary, the results of this study demonstrated that PRL and its receptor were expressed in NP tissues, and decreased concomitantly with the retardation of IVDD. We also demonstrated that IVDD induced by needle puncture in a rat model could be retarded by intradiscal injection of exogenous PRL. The protective effects of PRL were primarily mediated through the inhibition of inflammatory responses and apoptosis of NPCs via the NF-κB pathway. These findings might provide a novel therapeutic strategy for IVDD.

## Materials and methods

All human and animal procedures were approved by the Ethics Committee of the First Affiliated Hospital of Soochow University (Jiangsu, China).

### Human specimens

Human degenerated lumbar disc NP tissues were obtained from symptomatic patients undergoing microencoscopy discectomy (*n* = 5). MRI showed that all patients had disc space narrowing and hypointense NP. Non-degenerated disc NP tissues were obtained from the lumbar discs of scoliosis patients undergoing deformity correction surgery (*n* = 5).

### Experimental animals

A total of 80 3-month-old male Sprague-Dawley rats at osseous maturity^37^ (450 ± 50 g) were purchased from the Experimental Animal Center of Soochow University. The animals were kept in a ventilated environment with a 12:12 h light–dark cycle at a constant temperature of 21 °C.

### Operation procedures and groups

After 12 h of fasting and 4 h of water deprivation, all the animals were anesthetized with intraperitoneal injection (3.5 mL/kg) of 10% chloral hydrate (Sigma, St. Louis, MO, USA). An experimental model of IVDD was established by submitting the rats to percutaneous disc puncture with a 20-gauge needle on levels 7–8 (Co7–8) and 8–9 (Co8–9) of the coccygeal^38^. To ensure that degenerative effects could be produced, after the needle punctured the annulus fibrosus, it was rotated for 5 s and held for 30 s. To avoid the possible influences of individual differences, we defined rats punctured at discs Co7–8 as the PRL injection group (*n* = 80), those punctured at discs Co8–9 as the PBS injection vehicle group (*n* = 80), and rats with non-punctured discs at Co9–10 as the control group (*n* = 80). Furthermore, the PRL injection group was equally divided into low (10 μg/mL, *n* = 40) and high (100 μg/mL, *n* = 40) PRL concentration groups. The dosage of prolactin adopted in the current study was previously demonstrated to show nucleus pulposus cells survival effects in vivo. To eliminate the influences of the injected volume, only 2 μL PBS or high and low concentrations of PRL were injected into the center space of the NP at a depth of 6 mm^39^. All injections were performed with a 33-gauge Hamilton syringe (Hamilton Co., Reno, NV, USA), and this needle size did not induce degenerative disc changes as previously described^40^.

### X-ray and MRI examination

After 4, 7, 14, and 28 days of initial annulus puncture, 10 rats in each group (*n* = 10) were randomly chosen to undergo X-ray and MRI before they were sacrificed. These rats were kept in a supine position with their tails placed in a straight line on a molybdenum target radiographic image unit (GE Healthcare, Chicago, IL, USA). Radiographs were taken at a collimator-to-film distance of 66 cm, an exposure of 63 mAs, and a penetration power of 35 kV. MRI was performed using a 1.5T system (GE) to obtain T2-weighted images (repletion time, 3000 ms; echo time, 80 ms; field of view, 200 × 200 mm; slice thickness, 1.4 mm) in the coronal plane.

### Radiological analysis

All the radiographic images were saved in a medical imaging software system (DICOM3.0, Neusoft® PACS/RIS, Liaoning, China), and analyzed by an experienced radiologist blinded to the study. IVD height and the adjacent upper and lower vertebral body height were measured using the measuring tools of this software. The DHI was obtained from these values^41^. Changes in the DHI of experimental discs were expressed as %DHI compared to the values of normal discs. According to the modified Thompson classification^42^, the MRI images were classified as Grades I to IV (I, normal; II, minimal decrease of signal intensity but obvious narrowing of high signal area; III, moderate decrease of signal intensity; and IV, severe decrease of signal intensity) by assessing the signal intensity of the T2-weighted images.

### Histological and immunohistochemical analyses

The IVDs were collected after the rats were sacrificed, fixed in 10% formalin for 48 h, decalcified with 10% EDTA (Sigma) for 30 days, and embedded in paraffin wax. The paraffin blocks of IVD were cut into 5 μm coronal sections containing the endplate, AF, and NP, and then stained with either H&E or Safranin-O/Fast green. The histological grading scale system included four categories with scores ranging from 4 points (normal) to 12 points (serious degeneration) using the method established by Masuda^42^. To observe the specific expression of collagen type II (1:200), collagen type I (1:200), TNF-α (1:200), IL-1β (1:1000), cleaved-caspase 3 (1:100), p65 (1:500), IκBα (1:100), and IKKα (1:100) (rabbit anti-rat primary antibodies, Abcam, Shanghai, China) in the tissues, sections underwent antigen repair with proteases after de-waxing and gradient hydration. Then the tissues were blocked in 5% normal horse serum and incubated with the appropriate primary (300 μL per section) antibodies at 4 °C overnight. After rinsing, the tissue sections were incubated for 30 min with biotin-conjugated secondary antibodies and avidin-biotin enzyme reagents. Color was developed by incubating with the chromogen 3.3’-diaminobenzidine (DAB) tetrahydrochloride, followed by counterstaining with hematoxylin. TUNEL staining was performed using an in situ Cell Death Detection Kit (Roche, Indianapolis, IN, USA) to analyze the apoptosis of NPCs. After de-waxing, sections were incubated with proteinase K (15 μg/mL) for 15 min and then washed three times with PBS. Sections were incubated with the TUNEL reaction mixture for 60 min at 37 °C in a humidifying box. Then they were incubated with coverter-peroxidase solution at 37 °C for 30 min. After rinsing in PBS, the sections were incubated with DAB substrate for 5 min and stained with hematoxylin.

### Statistical analysis

All quantitative data are shown as the mean ± standard deviation. The two-tailed Student’s *t*-test was utilized to make comparisons between groups, and one-way analysis of variance was used to perform multiple comparisons. The effects of time and treatment on nonparametric data (MRI and histology grading for each parameter) were determined with the Kruskal-Wallis and Mann–Whitney U tests. Calculations were performed using SPSS version 17.0, and *P* values less than 0.05 were considered statistically significant.
